# Integration of communicable and non-communicable diseases within the health system of India: A window of opportunity?

**DOI:** 10.3389/fpubh.2022.1079827

**Published:** 2023-01-09

**Authors:** Sudip Bhattacharya, Shailesh Tripathi, Pratima Gupta, Saurabh Varshney, Vidisha Vallabh

**Affiliations:** ^1^Department of Community and Family Medicine, All India Institute of Medical Sciences (AIIMS), Deoghar, India; ^2^Rajendra Institute of Medical Sciences, Ranchi, Jharkhand, India; ^3^Department of Microbiology, All India Institute of Medical Sciences (AIIMS), Deoghar, India; ^4^Department of ENT (Otorhinolaryngology), All India Institute of Medical Sciences (AIIMS), Deoghar, India; ^5^Department of Community Medicine, Himalayan Institute of Medical Sciences, Dehradun, Uttarakhand, India

**Keywords:** infectious diseases, non-communicable disease (NCD), epidemiologic transition, hypertension, policy and guidelines, low and middle-income countries (LMIC)

## Introduction

Non-communicable diseases (NCDs) and communicable diseases (CDs) have an overlap of classic risk factors contributing to the double burden of diseases in India, which acts as a driver of epidemiological transition (1). A pseudo divide between CDs and NCDs was first noticed by Philip Sartwell, an eminent epidemiologist, more than 50 years ago. He expressed the opinion that the “separation of acute from chronic, or infectious from non-infectious is arbitrary,” and in reality, NCDs and CDs “differ superficially” (2). Sartwell formulated Sartwell's Law for the distribution of the incubation period of CDs, which was later adopted by epidemiologists to estimate the latent period of NCDs with an unknown etiology. This approach has, to a certain extent, blurred the boundaries between NCDs and CDs (3, 4). Despite adoption of the model for CDs, Sartwell's model has struggled to bridge the profound disease divide that is present between NCDs and CDs for epidemiologists and clinicians alike, which is termed the “unfortunate schism” by Reuel (5). Prominent epidemiologists, like Elizabeth Barrett-Connor, have supported the argument that the stream of NCDs and CDs has much familiarity and methods to offer to each other, arguing that NCDs and CDs are not “separate and unrelated species” (6). Her argument is strengthened by similar observations made in the LMICs by experts, organizations, and government guidelines that rally around the efforts to implement NCD and CD control and management. The LMICs have taken cognizance of the shared features of NCDs and CDs, such as continuing care and common high-risk populations, after their long-standing struggle with the dual burden of disease. There are also well-known direct interactions between certain CDs and cancer types/NCDs and vice versa. There is a growing body of evidence that indicate that India needs to focus on restructuring the health system and look for an inimitable prospect at breaking the boundaries of NCDs and CDs. The double burden of LMIC and NCD is putting tremendous pressure on the healthcare system in India. The sharply increasing prevalence of type II diabetes mellitus (T2DM) among municipal populations (5–15%), among semi-municipal populations (4–6%), and among countryside populations (2–5%), along with the highest incidence of tuberculosis (TB) worldwide (2.0 million cases each year), is usurping much of the resources needed for development (7, 8). To combat the dual burden of NCDs and CDs in India, a combined and multidisciplinary approach is the answer to prevent and control both NCDs and CDs. To draw an effective plan of action in India, it is imperative to analyze the determinants and distribution patterns of NCDs and CDs. Rapid and unplanned urbanization that rose along with the heralding of the Industrial Revolution also birthed the dual disease burden. Rural-to-urban migrants in India are some of the high-risk vulnerable groups who carry the risk factors in the dual burden of diseases. The poor working, eating, and living conditions make these migrants more vulnerable to the attack of chronic infections like TB (9). In addition to this scenario, it was observed that the transmission capability of CDs among the rural-to-urban migrants is higher than their native communities (10). Among this growing migrant population in the urban slums of India, the emergence of NCDs, namely, hypertension and type 2 diabetes mellitus, is quite common alongside the presence of CDs (11). “Reversal of the social gradient” is witnessed as NCDs, such as obesity, are now common among people living in slum areas, which was more prevalent earlier only among people belonging to higher socioeconomic strata (12). The coexisting NCDs and CDs in the slum area highlight the importance of identifying high-risk groups who could turn out to be victims of the dual disease burden in India. It will be interesting to examine whether the dual burden and its extent is “dose-dependent,” and what its linkage is with the sociodemographic profile of the population.

In multiple studies, the coexistence and predisposition of both NCDs and CDs in adult life have been attributed to the low birth weight of babies, which has been established as the environmental insult [“Barker's hypothesis” or Fetal origin of Adults Disease (FOAD)] (13). This life course phenomenon, *in utero* insult, and predisposition of both NCDs and CDs in later life are relevant to LMICs where multiple unfavorable conditions for living commonly coexist. However, CDs can represent early-life negligence and predispose to NCDs later on, including multiple types of cancer (14). NCD burden, especially cancer burden, is raising at a rapid pace in many LMICs due to longer life expectancies and changes in lifestyle patterns (15). There is growing evidence to suggest that various types of cancer associated with CDs impact the LMICs disproportionately (16). Cancers of the lung, the gastrointestinal (GI) tract, and the liver reveal the involvement of major infection-causing factors, and the LMICs are predominantly affected. Based on research findings, seropositivity with *Helicobacter pylori* for 10 years increases the chances of getting gastrointestinal (GI) cancer by 6 times (17). Similarly, liver cancer is associated with hepatitis B virus (HBV) and hepatitis C virus (HCV) infections (15). Another study revealed that the risk of developing Kaposi sarcoma (KS) is 1,000–5,000 times higher among patients who are immune–compromised (17). Thus, KS (an NCD) not only responds to the management of the CD but is also associated with the staging of the CD (18). Research on LMICs has also documented the association between the Epstein– Barr Virus (EBV) and the endemic Burkitt's lymphoma (BL) and has highlighted the association between EBV, BL, and malaria. These studies have also suggested alternative pathways of interaction between CDs and NCDs (19, 20). In India, it is also evident that a higher risk of NCD may perhaps put the population at elevated risk for common CDs and vice versa. Type 2 diabetes mellitus can interact with and complicate numerous CDs of public health importance like human immunodeficiency virus (HIV), TB, malaria, and many more. Patients with HIV on antiretroviral treatments can present with type 2 diabetes mellitus and lipid disorders and are at more risk of experiencing cardiovascular events due to adverse effects of the drugs. Evidence suggests that patients with type 2 diabetes mellitus have three-times more risk of active TB infection compared to those without non-diabetes (21). In India, two million incidences of TB cases annually have attributed 12.9% cases (~250,000) to type 2 diabetes mellitus alone. It is estimated that, in India, 16.0% of TB cases are attributable to type 2 diabetes mellitus (type 2 DM) (22). Issues related to CD and NCD co-morbidity like the population attributable risk (PAR) of type 2 diabetes mellitus among patients with TB and the outcome of TB in patients with diabetes must be resolved with future studies. In India, the lack of data and poor surveillance mechanism on co-morbidity limit the perceptive analysis of overlapping areas-gray zones of CDs and NCDs. The evidence mentioned above has established the urgency in concurrent surveillance of both NCD and CD morbidity like type 2 diabetes mellitus and TB. Co-morbidity data can help us to recognize the dual disease burden better and target harmonized care in India, where the dual disease burden is quite common. We expressed our opinion that, international organizations could play a pivotal role in the integration of NCD and CD supervision mechanisms within the global health context. Simultaneously, international agencies can facilitate to take up the dual supervision strategy at the national level that is customized to suit national and local needs. The amalgamation of varied dual disease surveillance data in India has demonstrated essential health gaps (23). The most important obstacle we are facing in India is that research on the incidence of infection-related cancer is still in the nascent stage. The strong association between CDs and cancers observed in developed countries underscores the vision of managing NCDs through low-cost interventions which are traditionally customized for CD management. Certainly, the prevention of cervical cancer, liver cancer, and human papillomavirus (HPV)-associated oro-genital cancer has followed this model, reaping benefits from economical yet effective interventions.

We believe that India has a window of opportunity to target public health services/programs like the National Programme for Prevention and Control of Cancer, Diabetes, Cardiovascular Diseases and Stroke (NPCDCS), and Integrated Disease Surveillance Programme (IDSP) to reach those populations who experience dual risks of CDs and NCDs and focus on their overlapping care requirement. It is important to focus on susceptible populations by improving access to health services and strengthening the social safety net. As an example, the occurrence of *H. Pylori infection* is closely connected to various socioeconomic factors such as poor education, poverty, overcrowding, and poor sanitation (24) and all these homogeneous risk factors also contribute to viral hepatitis, TB, type 2 DM, and chronic heart disease.

Public health approaches to tackle NCDs and CDs need to be parallel, rather than vertical. In the endemic areas of CDs and NCDs, aligning primary preventive measures could provide significant payback to reduce the dual disease burden/co-morbidity. Similarly, screening and diagnostics can be placed in line by skilled healthcare workers for preventing and treating both NCDs and CDs (25). However, the integration of both NCD and CD programs/management has the tendency to overstretch the already burdened Indian health system. So, the integration of NCD management can be done with existing primary care systems, which tends to put more emphasis on CDs (26). Additionally, we can think about the pooled supply systems at the international level, such as those on similar lines with vaccine procurement, which can be extended to include essential NCD drugs. Strategies for intervention/integrated approach for NCDs and CDs should consider underlying common relevant risk factors, such as environmental factors, sociodemographic factors, and screening at-risk individuals for co-morbidities, which could enable poor people to have access to direct treatment and long-term follow-up. The convergence of both NCDs and CDs is a natural phenomenon due to demographic transition; we have both opportunities to utilize and challenges to face so as to manage this dual disease burden in India. If we utilize the existing opportunities in the health system and purposefully draw a parallel with preventive, health-promotive, diagnostic, and treatment mechanisms for NCDs and CDs, the efforts could become a central point for the transformation of future public health policies.

## Author contributions

All authors listed have made a substantial, direct, and intellectual contribution to the work and approved it for publication.
